# Virulence of *Candida* Isolates in Patients with Tuberculosis and Oral/Oesophageal Candidiasis: Co-Infection Evaluation

**DOI:** 10.3390/jof11090665

**Published:** 2025-09-11

**Authors:** Rayana Larissa Pinheiro Soares Ferreira, Alessandra Teixeira Macedo, Conceição de Maria Pedrozo e Silva de Azevedo, Sirlei Garcia Marques, Marliete Carvalho Costa, João Carlos Maia Dornelas de Oliveira, Paulo Henrique Fonseca do Carmo, Yankee Costa Magalhães Diniz, Heylane Ferreira Cutrim, Cristina Andrade Monteiro, Maria Rosa Quaresma Bomfim, Daniel Assis Santos, Rodrigo Assuncao Holanda, Julliana Ribeiro Alves Santos

**Affiliations:** 1 Laboratório de Micologia, Universidade CEUMA (UNICEUMA), São Luís 65067-120, Maranhão, Brazil; rayana-larissa@hotmail.com (R.L.P.S.F.); macedo.alessandra@discente.ufma.br (A.T.M.); cmarliete@yahoo.com (M.C.C.); 2 Mestrado em Ciências da Saúde, Universidade Federal do Maranhão (UFMA), São Luís 65080-805, Maranhão, Brazil; conceicaopedrozo@gmail.com; 3 Laboratório CEDRO, Hospital Universitário da Universidade Federal do Maranhão (HU-UFMA), São Luís 65020-570, Maranhão, Brazil; sirleigmarques@gmail.com (S.G.M.); yankeecm@gmail.com (Y.C.M.D.); heylanecutrim@hotmail.com (H.F.C.); 4 Laboratório de Micologia, Departamento de Microbiologia, Universidade Federal de Minas Gerais (UFMG), Belo Horizonte 31270-901, Minas Gerais, Brazil; joaocmdo@yahoo.com.br (J.C.M.D.d.O.); dasufmg@gmail.com (D.A.S.); 5 Instituto de Biociências, Universidade Estadual Paulista (UNESP), Campus de Botucatu, Botucatu 18618-689, São Paulo, Brazil; 6 Instituto Federal de Educação, Ciência e Tecnologia do Maranhão (IFMA), São Luís 65080-805, Maranhão, Brazil; cristinamonteiro@ifma.edu.br; 7 Laboratório de Biologia Molecular de Microrganismos Patogênicos, Universidade CEUMA (UNICEUMA), São Luís 65075-120, Maranhão, Brazil; mrqbomfim@gmail.com; 8 Instituto de Ciências Biológicas, Universidade de Pernambuco (UPE), Recife 50100-130, Pernambuco, Brazil; julliana.rasantos@upe.br; 9 Departamento de Medicina Tropical, Centro de Ciências Médicas, Universidade Federal de Pernambuco (UFPE), Recife 50670-901, Pernambuco, Brazil

**Keywords:** antifungal treatment, *Candida*, co-infection, *Mycobacterium tuberculosis* complex, tuberculosis

## Abstract

Tuberculosis (TB) is an infection caused by *Mycobacterium tuberculosis* complex (MTBC), which can be exacerbated by fungal infections. This study evaluated the clinical characteristics and virulence of *Candida* spp. in patients with tuberculosis. Antifungal sensitivity, phospholipase and proteinase production, biofilm formation, phagocytic index, and reactive oxygen (ROS) and nitrogen (RNS) species were assessed. *Candida* spp. were isolated from 14 patients, 28.5% women and 71.4% men, mainly from sputum and tracheal secretions. Five (35.7%) patients were co-infected with *Mycobacterium*, *Candida*, and HIV. *Candida albicans* (78.6%) and *Candida tropicalis* (21.4%) were identified in all 14 patients. All isolates showed sensitivity to amphotericin B and dose-dependent responses to fluconazole (16 μg/mL). Phospholipase activity was detected in 35.7% of the isolates, whereas all isolates showed proteinase activity (100%). A significant difference in phospholipase activity, phagocytosis, and production of reactive oxygen species (ROS) and nitrogen species (RNS) was observed when *Candida* isolates from patients with TB, living with or without HIV, were compared to *Candida* isolates from healthy individuals. All isolates were biofilm producers. This study highlights the relevance of mycoses diagnosis in patients with TB, since *Candida* spp. may be more virulent and contribute to the deterioration of the clinical condition.

## 1. Introduction

In Brazil, approximately 69,000 new cases of tuberculosis (TB) were reported between 2016 and 2017. Brazil ranks 20th among the countries selected for the control of TB and 19th among the countries prioritised for treating TB-HIV co-infection [1]. Co-infection with *Mycobacterium tuberculosis* complex (MTBC) species and HIV has a synergistic effect [2]. However, the interactions between pathogenic fungi and MTBC in patients living with or without HIV remain unknown.

The fungal pathogen *Candida albicans* is a major cause of infection in humans and predominantly affects individuals with TB. However, the incidence of infections caused by non-*albicans* species has increased [3]. Oral candidiasis is an important clinical marker of TB, indicating the need for further laboratory monitoring of this co-infection [4]. *Candida* species have auxiliary mechanisms to adapt and multiply in the host. The ability of yeast to transition between buds, hyphae, and pseudohyphae is a major virulence factor [5].

Improper and late diagnoses of mycoses lead to the misuse of antibacterials and empirical use of antifungal agents. This contributes to the underdiagnosis of fungal diseases and emergence of resistant strains [6]. However, the diagnosis of fungal infections has recently received considerable attention owing to the occurrence of several pulmonary mycoses with symptoms similar to those of TB. Therefore, it is necessary to perform clinical tests to detect secondary fungal co-infections, avoid erroneous therapy, and prevent severe comorbidities [7].

The objective of our study was to describe the clinical characteristics of patients with tuberculosis and oral/oesophageal candidiasis, and to evaluate the virulence of *Candida* isolates obtained from patients infected with *M. tuberculosis* complex (MTBC)). The focus of our study was to assess whether *Candida* isolates obtained from patients with TB (whether or not they were living with HIV) exhibited increased virulence in cases of oral/oesophageal candidiasis.

## 2. Materials and Methods

### 2.1. Ethical Approval

The study was approved by the Research Ethics Committee of Universidade CEUMA, in accordance with the requirements of Resolution 466/12 of the National Health Council (opinion number: 1,570,408), and by the Ethics Committee on the Use of Animals (CEUA) of the Federal University de Minas Gerais—UFMG (approval number: 83/2019).

### 2.2. Study Population

This study included 38 patients admitted to a public reference hospital in São Luís, Maranhão, Brazil, between 2017 and 2018 for the diagnosis and treatment of TB. Patients suspected of having TB and candidiasis in the oral and/or oesophageal cavities were included in this study. These patients were clinically evaluated by a specialist in infectious diseases, and individuals with co-infection or MTBC/HIV/pathogenic fungi, and individuals co-infected with MTBC/pathogenic fungi were investigated. To confirm co-infection, individuals were assessed for the concurrent presence of MTBC and fungal infections.

### 2.3. Samples and Strains

Sputum, tracheal secretions, urine, and blood samples from patients with TB were used for fungal research through direct examination and identification by automated MALDI-TOF (Bruker, Billerica, MA, USA), followed by sequencing of the internal transcribed spacer (ITS) region. *Candida* isolates obtained from the oral mucosa of healthy individuals, i.e., without any signs or symptoms of infection, were used as the control group. The yeast isolates were cryopreserved at −80 °C in Sabouraud dextrose broth containing 20% glycerol. To activate them, the yeast isolates were grown on a Sabouraud dextrose agar (SDA) surface at 37 °C for 48 h.

### 2.4. Molecular Identification: DNA Isolation and Sequencing

DNA isolation was performed in accordance with the protocol described by Vieira et al. [8]. In brief, *Candida* isolates were grown on a Sabouraud dextrose agar (SDA) surface at 37 °C for 48 h. Approximately 50–100 mg of biomass was then resuspended in 500 μL of lysis buffer (250 mM Tris-HCl, 25 mM EDTA, 0.5% SDS, 250 mM NaCl, and pH 8.0) and vortexed for 1 min. The sample was then subjected to a heating (80 °C)/freezing (−20 °C) cycle for 20 min each. The samples were then centrifuged at 1077× *g* to harvest the supernatant. A 24:1 mixture of chloroform and isoamyl alcohol was added to the supernatant, followed by vortex homogenisation and centrifugation at 14,475× *g* for 10 min at 4 °C. The aqueous upper phase was harvested and then DNA was precipitated by adding 0.7 volumes of ice-cold isopropanol and centrifuging at 14,475× *g* for 30 min at 4 °C. The supernatant was then discarded and the DNA pellet washed with 70% ethanol (*v*/*v*), before being centrifuged again (14,475× *g* at 4 °C for 10 min). Finally, the DNA pellet was dried at room temperature and resuspended in 40 μL of sterile ultrapure water. Its concentration was then measured by spectrophotometry (NanoDrop, Thermo Fisher Scientific, Waltham, MA, USA) and the sample was stored at −20 °C.

The partial 18S-5.8S-28S ribosomal DNA (rDNA) loci were amplified by PCR using the modified primers ITS5 (5′-GGAAGTAAAAGTCGTAACAAGG-3′) and ITS4 (5′-TCCTCCGCTTATTGATATGC-3′) [8,9]. The 25 μL PCR cocktail underwent initial denaturation for 4 min at 94 °C, followed by 40 cycles (1 min at 94 °C, 1 min at 55 °C, and 2 min at 72 °C), and a final extension for 5 min at 72 °C. The amplicons were subjected to 1% agarose gel electrophoresis and stained with GelRed (Biothym, Fremont, CA, USA) for visualisation under an ultraviolet transilluminator. The amplicons were purified using the Wizard^®^ SV Gel and PCR Clean-Up System (Promega, Madison, WI, USA), and their concentrations were estimated by spectrophotometry (NanoDrop, Thermo Scientific). DNA sequencing was performed using the chain termination method (ABI3730 sequencer; Applied Biosystems, Foster City, CA, USA; polymer POP7 and BigDye v3.1) by the Myleus Biotechnology Company (Belo Horizonte, Brazil). The quality of the programs was calculated using Phred software (version 071220.b) parameters.

### 2.5. Inoculum Preparation

The fungal inoculum was prepared in sterile saline solution (0.9% NaCl, filtered to 0.22 μm) by measuring the transmittance using a spectrophotometer at 530 nm. This produced a transmittance of 75–77%, corresponding to 1 × 10^6^ to 5 × 10^6^ cells/mL.

### 2.6. Antifungal Susceptibility Testing

The minimum inhibitory concentrations (MICs) for fluconazole and amphotericin B (Sigma-Aldrich, St. Louis, MO, USA) were determined using microdilution test in RPMI-1640 medium, as proposed by the Clinical and Laboratory Standards Institute (CLSI) [10]. In microdilution tests, MIC for fluconazole was determined visually as 50% growth inhibition and classified as susceptible (≤8 μg/mL), dose-dependent sensitive (16–32 μg/mL), or resistant (≥64 μg/mL). For amphotericin B, the visual reading was determined as 100% growth inhibition, with the sample being classified as either sensitive (≤1 μg/mL) or resistant (>1 μg/mL).

### 2.7. Assessment of Virulence Factors

Phospholipase and proteinase activities were determined as previously described [11,12,13]. For the phospholipase test, the egg yolk was aseptically added to a previously sterilised agar medium base at 55 °C (22.8 g of NaCl, 0.22 g of CaCl_2_, 23.4 g SDA in 360 mL of distilled water). For the proteinase test, 60 mL of a sterilised bovine serum albumin (BSA) solution (0.5 g of BSA; 0.04 g of MgSO_4_·7H_2_O; 0.5 g of K_2_HPO_4_; 1 g of NaCl; 0.2 g of yeast extract; 4 g of glucose; pH 4.0; and 0.22 μm filtered) was added to 140 mL of previously sterilised SDA at 55 °C. Subsequently, inocula of 3 × 10^3^ viable yeast cells per drop (suspended in sterilised 0.85% NaCl saline solution) were dripped onto the surface of the culture media for the phospholipase and proteinase tests. Then, two Petri dishes for each test were incubated at 37 °C for seven days. The diameters of the yeast colonies and the surrounding halos were measured in both tests. The precipitation zone intensity (Pz) values for proteinases and phospholipases were calculated as the average ratios of the measured diameters [colony/(halo + colony)]. Enzymatic activity was classified as negative (Pz = 1), weak (0.9–0.99), moderate (0.80–0.89), high (0.70–0.79), or very high (<0.70).

Biofilm formation was evaluated as described by Shin et al. [14] in 96-well polystyrene microplates. First, the samples were seeded in SDA medium and incubated in an oven at 37 °C for 24 h. The isolates were then diluted in saline and adjusted to 0.5 on the McFarland scale, corresponding to 1–5 × 10^6^ cells per mL (CLSI, 2008 [10]). The plates were sequentially filled in triplicates. For the negative control, only 200 μL of medium was transferred to the wells, while for the remaining wells, 180 μL of medium plus 20 μL of fungal suspension in saline was transferred. The plates were then incubated at 37 °C for 48 h in an oven. Subsequently, the plates were washed twice with sterile saline, after which 200 μL of methanol was added to each well for 15 min to fix the samples. The plates were then washed twice with saline, after which 200 μL of violet crystal was added for staining. Finally, 250 μL of ethanol was added to wash the wells before they were subjected to spectrophotometry with a 550 nm filter to measure the absorbance of each well. Based on the optical density of the isolates (OD.i) and the negative control (OD.c), the isolates were classified into the following categories: non-producer: OD.i < OD.c; weak producer: OD.i ≤ 2 × OD.c; moderate producer: 2 × OD.c ≤ OD.i ≤ 4 × OD.c; and strong producer: 4 × OD.c.

### 2.8. Phagocytosis Assay and Quantification of Reactive Oxygen and Nitrogen Species

Differentiated murine bone marrow macrophages were used for the phagocytosis assays [15]. First, 6- to 8-week-old healthy male C57BL/6 mice were anaesthetised (80 mg/kg ketamine hydrochloride and 15 mg/kg xylazine) and euthanised to aseptically remove the femurs and tibiae. The red marrow was then extracted by injecting 2–5 mL of RPMI 1640 medium into the medullary cavity from the epiphyses using a syringe with a 26G needle, followed by aspiration of the contents into ice-cold RPMI 1640 medium in a 50 mL conical bottom tube. The cells were then centrifuged at 300× *g* for 10 min at 4 °C and the supernatant was discarded. The cells were resuspended in supplemented RPMI 1640 medium containing 30% L929 growth-conditioning media, 20% foetal bovine serum, 2 mM glutamine, 100 units/mL penicillin–streptomycin, and 50 μM 2-mercaptoethanol, and their concentration was determined in a Neubauer chamber. An inoculum of 7 × 10^6^ cells was added to each Petri dish containing supplemented RPMI 1640 medium. The dishes were then incubated at 37 °C with 5% CO_2_ for seven days. The supplemented medium was changed every 48 h, and non-adherent cells were discarded. After seven days of incubation, immunophenotyping was performed using flow cytometry (FACSCantoII, Becton Dickinson, San Diego, CA, USA) to confirm differentiation into macrophages (CD11b+F4/80+). The supernatant was then discarded, and the attached cells were washed twice with sterile phosphate-buffered saline (PBS 1X; pH 7.2; filtered to 0.22 μm) before being transferred to a 50 mL conical bottom tube containing supplemented RPMI 1640 medium. An inoculum of 2 × 10^5^ cells was seeded in supplemented RPMI 1640 medium in a 24-well flat-bottom plate, with each well containing a circular coverslip (diameter 13–16 mm) to facilitate cell adhesion. The plates were then incubated at 37 °C and 5% CO_2_ for 18 h. *Candida* isolates, which had been grown on SDA at 37 °C for 48 h, were washed twice with sterile PBS. Then, 10^5^ yeast cells, suspended in supplemented RPMI 1640 medium, were added to each well for co-culture (5:1 yeast–macrophage ratio). The plates were then incubated at 37 °C and 5% CO_2_ for three hours. Coverslips were carefully removed, washed twice with sterile PBS, fixed with ice-cold methanol and stained with panoptic dye. The fungus–macrophage interaction (i.e., cell-surface interaction and internalisation of the fungus by the macrophage) was visualised by optical microscopy (Zeiss Axio Imager Z2, ZEISS, Oberkochen, Germany) to determine the phagocytosis index and the yeast-to-hyphae transition.

Under similar conditions, yeast–macrophage co-cultures (10^4^ yeast to 2 × 10^4^ macrophages) were grown in a 96-well flat-bottom plate to quantify reactive oxygen and nitrogen species (ROS and RNS as peroxynitrite (ONOO^−^), respectively) [16]. The cells were co-cultured in RPMI 1640 without phenol red at 37 °C and 5% CO_2_ for 3 and 24 h. Macrophages and yeast were also cultured individually per well. Then, the fluorescent dyes 20nM dihydrorhodamine 123 (DHR 123; Invitrogen, Life Technologies, Carlsbad, CA, USA) and 2,7-dichlorofluorescein diacetate (DCFH-DA; Invitrogen, Life Technologies) were added to each well for detection of ROS and RNS, respectively. The microplate was then incubated at 37 °C for 30 min and the fluorescence was quantified using a spectrophotometer (Varioskan Flash, Thermo, Waltham, MA, USA) with an excitation wavelength of 485 ηm and an emission wavelength of 530 ηm. For resultant quantification of reactive species (RS, either for ROS or RNS) produced during phagocytosis and individually by the phagocytes or the fungi, the following formula was used: ${RS}_{resultant}={RS}_{co\text{-}culture}-({RS}_{phagocyte}+{RS}_{fungus})$. The assays were performed in sextuplicate. Data are expressed as arbitrary fluorescence units (AU) ± SEM.

### 2.9. Statistical Analysis

The results are presented as the mean ± standard deviation. Statistical analyses were performed using GraphPad Prism (version 5.0, GraphPad Software, San Diego, CA, USA). Non-parametric *t*-test and analysis of variance (ANOVA) were used. Differences were considered significant at the 95% confidence level (*p* < 0.05).

## 3. Results

### 3.1. Clinical and Laboratory Characterisation of TB/Candida/HIV Co-Infection

Of the 38 patients with TB who were assessed, 18 (47.3%) tested negative for *Candida* infection, 14 (36.8%) tested positive and 6 (15.7%) produced inconclusive results. The 14 *Candida* spp. samples (12 from sputum and 2 from tracheal sections) were evaluated for their clinical and laboratory characteristics (Table 1).

Fourteen patients had oral and/or oesophageal candidiasis. Co-infection with TB, *Candida*, and HIV was observed in 35.7% of the patients. Of these patients, 28.5% were women and 71.4% were men, with an average age of 38.2 years (Table 1).

### 3.2. Sequencing of the Partial 18S-5.8S-28S Ribosomal DNA (rDNA) Loci

The clinical isolates were identified as *C. albicans* (78.6%) and *C. tropicalis* (21.4%) by sequencing the partial 18S-5.8S-28S ribosomal DNA (rDNA) loci. These results were confirmed by MALDI-TOF (Table 1). In the control group, 58.3% of the isolates were identified as *C. albicans*, whereas the remaining 41.7% consisted of *C. orthopsilosis*, *C. tropicalis*, *C. digboiensis*, and *C. parapsilosis* (Supplementary Table S1).

### 3.3. Antifungal Susceptibility Profile

Only clinical isolate 6 (*C. tropicalis*) exhibited dose-dependent sensitivity (S-DD) to fluconazole; the other isolates were sensitive to azole (MIC ≤ 16 μg/mL). The MIC for amphotericin B varied from 0.25 to 1 μg/mL as the samples were sensitive to polyene (see Table 2).

### 3.4. Virulence Factors

**Figure 1:**
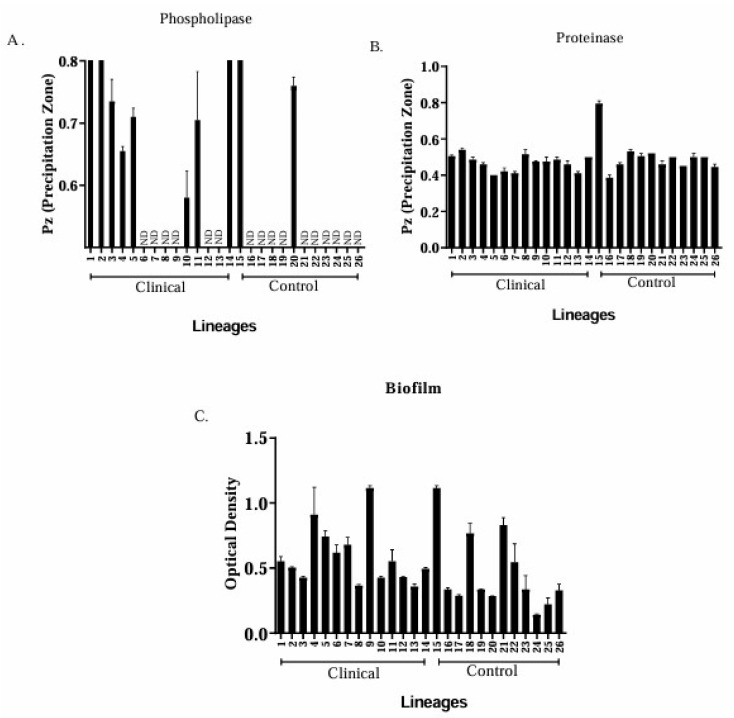
Intensity of the precipitation zone (Pz) for clinical isolates and controls in phospholipase (**A**) and proteinase (**B**) tests. The biofilm formation ability of *Candida* species was assessed using optical density (OD) (**C**). ND: not detected.

Among the clinical isolates, 35.7%, 21.45%, and 42.8% exhibited high, very high, and no phospholipase activity, respectively. In contrast, 83% of the isolates in the control group did not show phospholipase activity, whereas only two isolates showed moderate (8.3%) and high (8.3%) activities (Figure 1A). Proteinase production was high in all clinical isolates (100%), whereas in the control group, 91.6% and 8.3% of the isolates showed very high and moderate activity, respectively (Figure 1B). Clinical isolates showed good biofilm-forming ability; however, this ability varied among the isolates. Isolate 4 (*C. albicans*; OD = 0.911) showed the highest capacity for biofilm formation, whereas isolate 9 (OD = 0.150) showed the lowest capacity (Figure 1C). In the control group, isolate 15 showed the highest production, with an OD of 1.114 (Figure 1C).

### 3.5. Phagocytosis Assay

**Figure 2:**
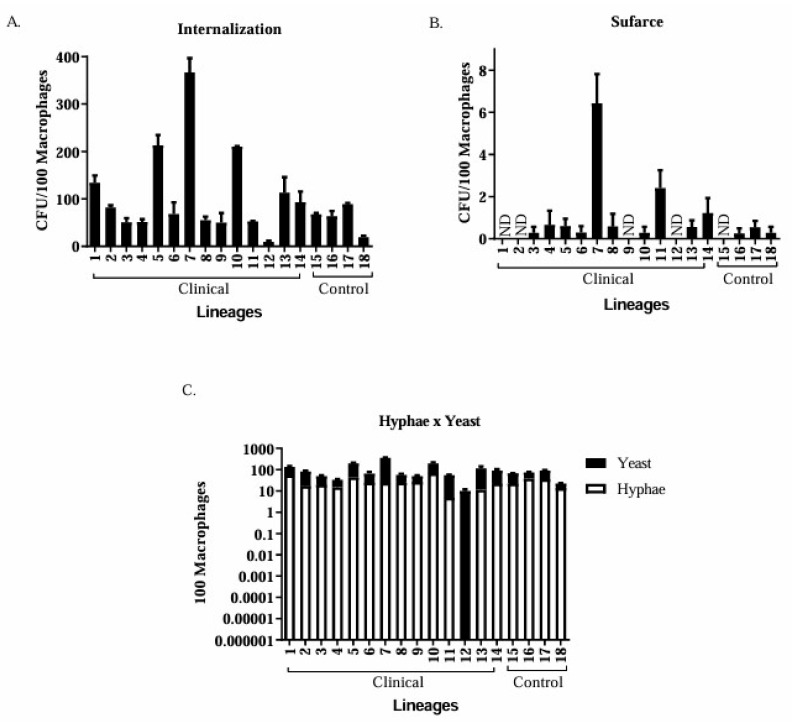
Fungus–macrophage interaction after three hours of co-culture. The phagocytic index was calculated by counting the amount of fungus internalised by macrophages (**A**). Surface fungus–macrophage interaction (**B**) and the yeast-to-hyphae transition were also determined (**C**). Data represent the mean of two independent experiment. ND: not detected.

The phagocytic index was calculated as the percentage of macrophages that internalised the fungus. An incubation time of 3 h was sufficient to promote fungal internalisation and hyphal and pseudohyphal production. Isolate 7 (*C. tropicalis*) exhibited the greatest internalisation (Figure 2A), along with an increase in macrophage surface area (Figure 2B). This isolate also had the highest significant average number of internalised yeasts (366 yeasts), being the most phagocytised when compared to the other clinical isolates that obtained an average of less than 300, and the control group had less than 100 internalised yeasts.

Isolate 7 exhibited the highest number of yeast on the macrophage surface, whereas the control group exhibited an average of less than one yeast. The production of hyphae was not detected for isolate 12, whereas isolate 1 produced significantly more hyphae than the control group isolates; this difference was significant (*p* < 0.05). Four clinical isolates (1, 2, 9, and 12) and one control isolate (15) showed no yeast cells on their surfaces (Figure 2B). Hyphal production was observed in 92.8% of isolates (Figure 2C).

### 3.6. Production of ROS and RNS by Murine Macrophages

**Figure 3:**
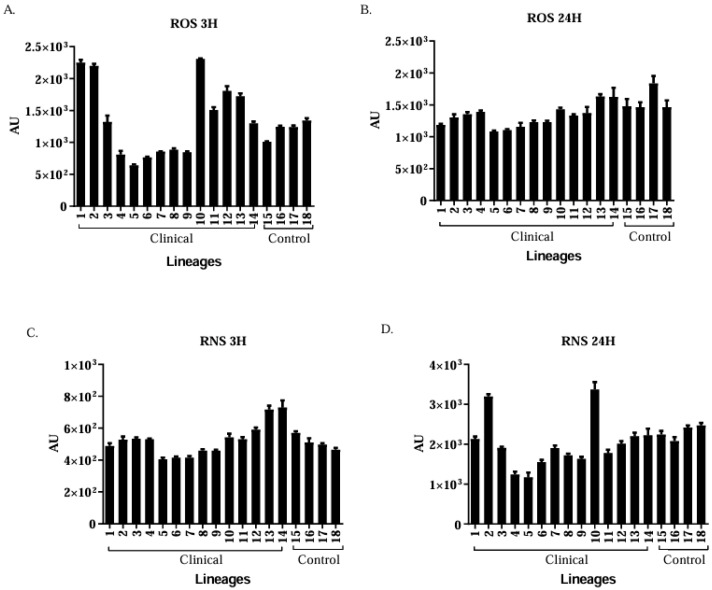
Quantification of ROS and RNS production for 3 h (**A**,**C**) and 24 h (**B**,**D**). AU: arbitrary units of fluorescence.

The highest average ROS (3 h) and RNS (3 h) production by macrophages was observed against clinical isolate 10 (*C. albicans*) compared to isolate 14 (see Figure 3A,B). Isolate 10 showed the highest RNS production after 24 h of phagocytosis (see Figure 3C,D).

#### Clinical Isolates of *Candida* spp. Are More Virulent than the Control Group Isolates

The means of the enzymatic activities and phagocytosis profiles of the clinical isolates and controls were compared using the Student’s *t*-test. Significant differences (*p* < 0.05) were observed between the two groups in phospholipase activity, yeast internalisation, and ROS and RNS production after 24 h ( Supplementary Table S2).

## 4. Discussion

In 2015, the World Health Organization proposed the strategy “For the End of Tuberculosis” with the aim of eradicating the disease by the year 2035. The goals are to prevent TB and provide integrated care to the patient, including early diagnosis, treatment, and management of other co-morbidities to reduce the incidence of the disease [17].

The incidence of HIV infection has also increased in patients with TB, which is commonly described as a co-epidemic [18]. Other co-infections in patients with TB have been documented in previous studies, including *M. tuberculosis* and *Cryptococcus gattii*, as well as *M. tuberculosis* and *Candida* spp. [19,20]. In the present study, five (35.7%) patients were co-infected with *Mycobacterium*, *Candida*, and HIV, featuring a triple co-infection. This type of infection is of concern because fungal infections can accelerate the clinical course of TB and aggravate the patient’s symptoms [21]. The results showed a higher prevalence of co-infection among men (71.4%), corroborating the study by Amiri et al. [22], in which co-infections of TB with fungi were more prevalent among men.

*Candida* spp. are commensals that are a part of the microbiota of healthy individuals. However, a weakened host immune system can cause these species to become pathogenic [23], especially when they express virulence factors such as proteolytic enzymes and are able to form biofilms. Although *C. albicans* is the most prevalent yeast in oral infections, the prevalence of non-*albicans* species such as *C. tropicalis*, *C. parapsilosis*, *C. glabrata*, *C. krusei*, and *C. dubliniensis* has also been reported [24]. Our samples were represented by *C. albicans* and *C. tropicalis*, corroborating the findings of Kali et al. [25], who reported the prevalence of *C. albicans* followed by *C. tropicalis* in samples from patients with pulmonary TB. Astekar et al. [3] revealed that *C. albicans* is present in the sputum of more than 50% of patients with pulmonary TB and is responsible for secondary infections in these patients.

Regarding the susceptibility profiles of the evaluated antifungals, only one isolate (*C. tropicalis*) showed dose-dependent sensitivity to fluconazole. According to Latha et al. [26], there are differences in the susceptibility of non-*albicans* species to antifungals compared to that of *C. albicans*.

In our study, the majority of *C. albicans* isolates were phospholipase producers. Only two isolates from the control group were phospholipase producers. *Candida* spp. exhibit significant phospholipase-producing capacity. All isolates were biofilm producers. Hasan et al. described *C. albicans* as the species with the highest production of biofilms that are more complex in composition than those of other species [27].

Several immune cells control fungal infections and macrophages are responsible for eliminating pathogens via phagocytosis [28]. Phagocytosis occurred in all isolates; however, hyphae were frequently produced, occurring in 92.8% of the clinical isolates. This demonstrates that the production of hyphae by *C. albicans* allows the yeast to survive the phagocytic actions of host cells. Changes in yeast morphology enable the fungus to perforate macrophage membranes and escape phagocytosis. ROS and RNS production capacities were significantly higher in the control group than in the group represented by clinical isolates [29].

ROS and RNS are released to destroy the phagocytosed microorganisms. However, these microorganisms have mechanisms to circumvent phagocytosis, such as neutralisation of ROS and adaptation to phagolysosomes. Previous studies have shown that *C. glabrata* can survive and multiply within macrophages [30]. The multiplication and formation of hyphae within murine macrophages were also observed in our phagocytosis assays.

One limitation of this study was the small sample size of 14 isolates obtained from patients with TB and candidiasis. This number may reflect the clinical reality as well as the under-reporting of cases due to the absence of specific protocols for investigating fungi concomitantly in patients with TB. However, this quantity allowed us to compare the virulence factors of the isolates, providing new insights and highlighting the importance of evaluating co-infections.

## 5. Conclusions

This study highlights the importance of diagnosing mycoses in patients with TB as *Candida* spp. may be more virulent and contribute to the deterioration of the patient’s condition. Furthermore, this study is a pioneer in the investigation of TB/fungal co-infection in patients from a Brazilian state in the north-eastern region of the Amazon rainforest. The identification of *Candida* species in patients with TB is crucial for improving treatment and prognosis.

## Figures and Tables

**Table 1:** Clinical and laboratory characterisation of fungal isolates obtained from patients with TB and candidiasis with or without HIV co-infection.

Isolated	Gender	Age	Tuberculosis	Fungi Isolation	Sample	Identification by MALDI-TOF	HIV
1	F	38	+	+	Sputum	*C. albicans*	-
2	M	20	+	+	Sputum	*C. albicans*	-
3	F	28	+	+	Sputum	*C. albicans*	-
4	F	43	+	+	Sputum	*C. albicans*	-
5	M	43	+	+	Sputum	*C. albicans*	-
6	M	37	+	+	Sputum	*C. tropicalis*	-
7	M	77	+	+	Sputum	*C. tropicalis*	-
8	M	24	+	+	Tracheal Secretion	*C. albicans*	-
9	M	47	+	+	Tracheal Secretion	*C. albicans*	-
10	M	54	+	+	Sputum	*C. albicans*	+
11	F	23	+	+	Sputum	*C. albicans*	+
12	M	37	+	+	Sputum	*C. tropicalis*	+
13	M	44	+	+	Sputum	*C. albicans*	+
14	M	41	+	+	Sputum	*C. albicans*	+

Abbreviations: M, male; F, female; +, positive; -, negative. Age in years.

**Table 2:** Minimum inhibitory concentration (MIC) of fluconazole and amphotericin B against 14 clinical isolates (1–14) obtained from patients with TB and 12 control isolates (15–26) obtained from healthy individuals.

Isolated	FLU_50_	AmB_100_
1 (Cl)	4 (S)	0.5 (S)
2 (Cl)	2 (S)	0.5 (S)
3 (Cl)	4 (S)	0.5 (S)
4 (Cl)	4 (S)	0.5 (S)
5 (Cl)	1 (S)	1 (S)
6 (Cl)	16 (SDD)	1 (S)
7 (Cl)	2 (S)	1 (S)
8 (Cl)	4 (S)	1 (S)
9 (Cl)	1 (S)	1 (S)
10 (Cl)	4 (S)	0.5 (S)
11 (Cl)	4 (S)	1 (S)
12 (Cl)	4 (S)	2 (R)
13 (Cl)	4 (S)	1 (S)
14 (Cl)	2 (S)	0.5 (S)
15 (Co)	8 (S)	0.5 (S)
16 (Co)	1 (S)	1 (S)
17 (Co)	4 (S)	1 (S)
18 (Co)	4 (S)	1 (S)
19 (Co)	4 (S)	1 (S)
20 (Co)	4 (S)	1 (S)
21 (Co)	4 (S)	2 (S)
22 (Co)	8 (S)	0.5 (S)
23 (Co)	0.5 (S)	2 (R)
24 (Co)	2 (S)	1(S)
25 (Co)	2 (S)	1 (S)
26 (Co)	2 (S)	1 (S)

Abbreviations: FLU, fluconazole; AmB, amphotericin B; S, susceptible; SDD, dose-dependent sensitivity; R, resistant, Co, control; Cl, clinical.

## Data Availability

The original contributions presented in this study are included in the article/Supplementary Materials. Further inquiries can be directed to the corresponding author.

## Supplementary Materials

The following supporting information can be downloaded at https://www.mdpi.com/article/10.3390/jof11090665/s1, Table S1: Genetic identification of 14 clinical isolates (1–14) obtained from patients with tuberculosis and 12 control isolates (15–26) obtained from healthy individuals.; Table S2: Clinical isolates of *Candida* spp. are more virulent than those of the control group.
