# Supplementary material for: Virulence of Candida Isolates in Patients with Tuberculosis and Oral/Oesophageal Candidiasis: Co-Infection Evaluation
Source: J Fungi (Basel). 2025 Sep 11;11(9):665. doi: 10.3390/jof11090665 (PMC12470509; doi:10.3390/jof11090665)
Supplement: Supplementary file 1 [file jof-11-00665-s001.zip › jof-3758451-supplementary.pdf]

Journal of *Fungi*

Type of the Paper (Article.)

Virulence of *Candida* isolates in patients with tuberculosis and oral/oesophageal candidiasis: co-infection evaluation

Rayana Larissa Pinheiro Soares Ferreira [1], Alessandra Teixeira Macedo [1,2], Conceição de Maria Pedrozo e Silva de Azevedo [2], Sirlei Garcia Marques [3], Marliete Carvalho Costa [1], João Carlos Maia Dornelas de Oliveira [4], Paulo Henrique Fonseca do Carmo [5], Yankee Costa Magalhães Diniz [3], Heylane Ferreira Cutrim [3], Cristina Andrade Monteiro [6], Maria Rosa Quaresma Bomfim [7], Daniel Assis Santos [4], Rodrigo Assuncao Holanda [8,9*], and Julliana Ribeiro Alves Santos [8]

1 Laboratório de Micologia – Universidade CEUMA (UNICEUMA), São Luís, Maranhão, Brasil., rayanalarissa@hotmail.com; macedo.alessandra@discente.ufma.br; cmarliete@yahoo.com
2 Mestrado em Ciências da Saúde – Universidade Federal do Maranhão (UFMA), São Luís, Maranhão, Brasil. conceicaopedrozo@gmail.com
3 Laboratório CEDRO and Hospital Universitário da Universidade Federal do Maranhão (HU-UFMA) – São Luís, Maranhão, Brasil.; sirleigmarques@gmail.com; yankeecm@gmail.com; heylanecutrim@hotmail.com
4 Laboratório de Micologia – Departamento de Microbiologia – Universidade Federal de Minas Gerais (UFMG), Belo Horizonte, Minas Gerais, Brasil.; joaocmdo@yahoo.com.br; dasufmg@gmail.com
5 Instituto de Biociências da Universidade Estadual Paulista (Unesp), campus de Botucatu, Botucatu, São Paulo, Brazil; paulofonsecca@gmail.com
6 Instituto Federal de Educação, Ciência e Tecnologia do Maranhão (IFMA), São Luís, Maranhão, Brasil.; cristinamonteiro@ifma.edu.br
7 Laboratório de Biologia Molecular de Microrganismos Patogênicos – Universidade CEUMA (UNICEUMA), São Luís, Maranhão, Brasil.; mrqbomfim@gmail.com
8 Instituto de Ciências Biológicas– Universidade de Pernambuco (UPE), Recife, Pernambuco, Brasil; raholanda@yahoo.com.br; julliana.rasantos@upe.br
9 Departamento de Medicina Tropical, Centro de Ciências Médicas– Universidade Federal de Pernambuco (UPE), Recife, Pernambuco, Brasil; raholanda@yahoo.com.br
* Correspondence: raholanda@yahoo.com.br

Supplementary Materials:

Table S1: Genetic identification of 14 clinical isolates (1–14) obtained from patients with tuberculosis and 12 control isolates (15–26) obtained from healthy individuals.

Isolated	Species	Bit-score	E-value	Identification	GenBank Comparison Sequence Access
1	*Candida albicans*	983	0.0	100%	CP025165.1
2	*Candida albicans*	976	0.0	99%	KF241848.1
3	*Candida albicans*	985	0.0	100%	KU987839.1
4	*Candida albicans*	985	0.0	100%	CP025165.1
5	*Candida albicans*	987	0.0	99%	LC317484.1
6	*Candida tropicalis*	965	0.0	100%	EF197999.1
7	*Candida tropicalis*	957	0.0	100%	MG720231.1
8	*Candida albicans*	990	0.0	99%	CP025165.1
9	*Candida albicans*	597	7e-167	99%	MF797746.1
10	*Candida albicans*	989	0.0	100%	KY101883.1
11	*Candida albicans*	992	0.0	99%	CP025165.1
12	*Candida tropicalis*	854	0.0	97%	MG599209.1
13	*Candida albicans*	1000	0.0	99%	CP025165.1
14	*Candida albicans*	987	0.0	100%	CP025165.1
15	*Candida albicans*	913	0.0	99%	KJ651877.1
16	*Candida albicans*	1029	0.0	99%	CP025165.1

 https://doi.org/10.3390/jof11090665

17	ND	ND	ND	ND	ND
18	*Candida albicans*	994	0.0	99%	CP025165.1
19	*Candida orthopsilosis*	874	0.0	100%	KY102274.1
20	*Candida digboiensis*	678	0.0	100%	FJ011540.1
21	*Candida tropicalis*	508	4e-140	92%	KY711241.1
22	*Candida albicans*	719	0.0	100%	MK307750.1
23	*Candida parapsilosis*	957	0.0	100%	LC389772.1
24	*Candida albicans*	924	0.0	98%	CP025165.1
25	*Candida albicans*	950	0.0	99%	CP032012.1
26	*Candida albicans*	656	0.0	100%	MF797781.1

Abbreviations: ND, not detected.

Table S2: Clinical isolates of *Candida* spp. are more virulent than those of the control group.

**Clinical Isolates x Control**	**P value**
Phospholipase	0.0051 *
Proteinase	0.0900
Biofilm	0.3849
Internalization	0.0436*
Surface fungus-macrophage interaction	0.1532
Hyphae present in 100 counted macrophages	0.3727
Yeast present in 100 counted macrophages	0.0116*
ROS 3 horas	0.1257
ROS 24 horas	0.0004*
RNS 3 horas	0.4045
RNS 24 horas	0.0401*

Abbreviations: ROS, reactive oxygen species; RNS, reactive nitrogen species. * $p < 0.05$.
